# Two New Spiro-Heterocyclic *γ*-Lactams from A Marine-Derived *Aspergillus fumigatus* Strain CUGBMF170049

**DOI:** 10.3390/md17050289

**Published:** 2019-05-14

**Authors:** Xiuli Xu, Jiahui Han, Yanan Wang, Rui Lin, Haijin Yang, Jiangpeng Li, Shangzhu Wei, Steven W. Polyak, Fuhang Song

**Affiliations:** 1 School of Ocean Sciences, China University of Geosciences, Beijing 100083, China; 15632779760@163.com (J.H.); nancywang0410@163.com (Y.W.); linrui520@126.com (R.L.); yanghaijin52@163.com (H.Y.); cuculjp2016@163.com (J.L.); wert0715@163.com (S.W.); 2 CAS Key Laboratory of Pathogenic Microbiology and Immunology, Institute of Microbiology, Chinese Academy of Sciences, Beijing 100101, China; 3 School of Pharmacy and Medical Sciences, University of South Australia, Adelaide 5000, Australia; steven.polyak@unisa.edu.au

**Keywords:** marine-derived *Aspergillus fumigatus*, spiro-heterocyclic *γ*-lactam, cephalimysins

## Abstract

Two new spiro-heterocyclic *γ*-lactam derivatives, cephalimysins M (**1**) and N (**2**), were isolated from the fermentation cultures of the marine-derived fungus *Aspergillus fumigatus* CUGBMF17018. Two known analogues, pseurotin A (**3**) and FD-838 (**4**), as well as four previously reported helvolic acid derivatives, 16-*O*-propionyl-16-*O*-deacetylhelvolic acid (**5**), 6-*O*-propionyl-6-*O*-deacetylhelvolic acid (**6**), helvolic acid (**7**), and 1,2-dihydrohelvolic acid (**8**) were also identified. One-dimensional (1D), two-dimensional (2D) NMR, HRMS, and circular dichroism spectral analysis characterized the structures of the isolated compounds.

## 1. Introduction

**Figure 1:**
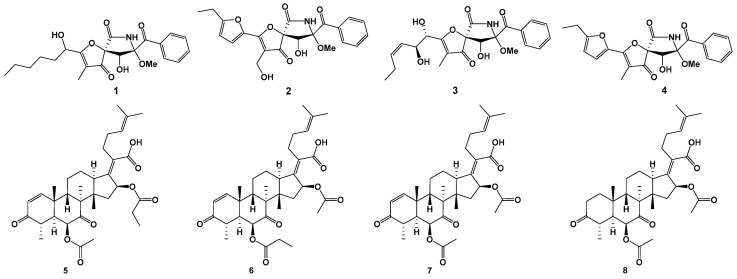
Chemical structures of **1**–**8**.

Marine-derived fungi are important resources of structurally and biologically diverse secondary metabolites in drug discovery [1,2,3,4,5,6]. A series of novel marine natural compounds have been isolated from marine-derived fungi of *Aspergillus fumigatus* strains, such as *E*-β-*trans*-5,8,11-trihydroxybergamot-9-ene and β-*trans*-2β,5,15-trihydroxybergamot-10-ene [7], diketopiperazines [8,9], indole alkaloids [10], fumigaclavine C [11], fumiquinazoline K [12], and gliotoxin analogues [13]. During our ongoing efforts to search for new bioactive metabolites from marine-derived fungi, an *Aspergillus fumigatus* strain CUGBMF170049 was isolated from a sediment sample that was collected from the Bohai Sea, China. Chemical investigations on an EtOAc-MeOH extracted fraction of its solid fermentation cultures resulted in the characterization of two new spiro-heterocyclic *γ*-lactam derivatives, cephalimysins M (**1**) and N (**2**), along with two known analogues, pseurotin A (**3**) [14], and FD-838 (**4**) [15], as well as four previously reported helvolic acid derivatives, 16-*O*-propionyl-16-*O*-deacetylhelvolic acid (**5**), 6-*O*-propionyl-6-*O*-deacetylhelvolic acid (**6**) [16], helvolic acid (**7**), and 1,2-dihydrohelvolic acid (**8**) [17] (Figure 1). Herein, we report the isolation and structural determination of the new compounds **1** and **2**. The antibacterial activities of the compounds were also investigated against a panel of both Gram-positive and Gram-negative bacteria, *Mycobacterium* bovis bacillus Calmette Guérin (BCG) and *Candida albicans*. Compounds **5**–**7** showed significant antibacterial activities against both *Staphylococcus aureus* and methicillin resistant *S. aureus*.

## 2. Results

### 2.1. Structure Elucidation

**Figure 2:**
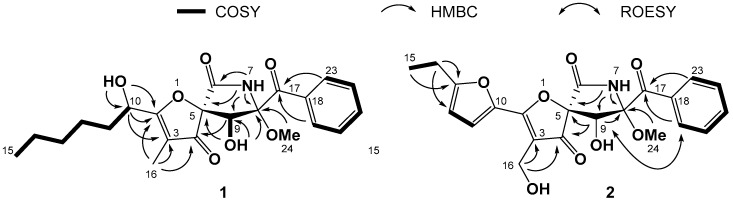
Key two-dimensional (2D) NMR correlations for **1** and **2**.

Compound **1** was obtained as pale yellow amorphous powder. Its molecular formula was determined as C_22_H_27_NO_7_ by HRESIMS *m/z* 440.1684 [M + Na]^+^ (calcd. for C_22_H_27_NO_7_Na 440.1680, Δmmu + 0.4) (Figure S1 in Supplementary Materials), which accounted for ten degrees of unsaturation. The ^1^H, ^13^C, and HSQC NMR spectra (Figures S2–S4 in Supplementary Materials) of compound **1** showed signals of two ketone carbonyls at *δ*_C_ 196.7 (C-4) and 196.4 (C-17), one amide carbonyl at *δ*_C_ 166.4 (C-6), a mono-substituted benzene ring (*δ*_C_ 133.4, *δ*_C_ 130.3/*δ*_H_ 8.25, *δ*_C_ 128.4/*δ*_H_ 7.53, *δ*_C_ 133.9/*δ*_H_ 7.64, *δ*_C_ 128.4/*δ*_H_ 7.53, 130.3/*δ*_H_ 8.25), two sp^2^ quaternary carbons at *δ*_C_ 188.5 (C-2) and 109.6 (C-3), four sp^3^ methylenes (*δ*_C_ 34.4/*δ*_H_ 1.64, *δ*_C_ 31.1/*δ*_H_ 1.24, *δ*_C_ 24.1/*δ*_H_ 1.37, and δ_C_ 22.0/*δ*_H_ 1.24), two sp^3^ oxygenated methines (*δ*_C_ 74.9/*δ*_H_ 4.38 and *δ*_C_ 67.0/*δ*_H_ 4.50), two sp^3^ oxygenated quaternary carbons at *δ*_C_ 91.2 (C-5) and *δ*_C_ 92.5 (C-8), and three methyls (*δ*_C_ 13.9/δ_H_ 0.84, *δ*_C_ 5.4/*δ*_H_ 1.64, and *δ*_C_ 51.7/*δ*_H_ 3.24) (Table 1). A comparison of ^1^H and ^13^C NMR data for compound **1** (Table 1) with those of previously reported pseurotin A (**3**) [14] revealed many similarities in their chemical structures, except for the oxygenated unsaturated side chain of **3** had been substituted with an oxygenated saturated fatty chain in **1**. Compound **1** had the same skeleton as that of pseurotin A, while the side chain of **1** is an oxygenated saturated fatty chain. The COSY correlations (Figure 2, and Figure S5 in Supplementary Materials) from H-19 (*δ*_H_ 8.25) to H-23 (*δ*_H_ 8.25), through H-20 (*δ*_H_ 7.53), H-21 (*δ*_H_ 7.64) and H-22 (*δ*_H_ 7.53) identified the mono-substituted benzene ring. Likewise, the fatty acid side chain was confirmed by COSY correlations from H-10 (*δ*_H_ 4.50) to H_3_-15 (*δ*_H_ 0.84), through H-11 (*δ*_H_ 1.64), H-12 (*δ*_H_ 1.37), H-13 (*δ*_H_ 1.24), and H-14 (*δ*_H_ 1.24). The lactam ring was evidenced by the HMBC correlations (Figure 2, and Figure S6 in Supplementary Materials) from H-9-OH (*δ*_H_ 6.34) to C-5 (*δ*_C_ 91.2), C-8 (*δ*_C_ 92.5), and C-9 (*δ*_C_ 74.9), as well as from H-7-NH (*δ*_H_ 9.90) to C-5 (*δ*_C_ 91.2), C-6 (*δ*_C_ 166.4), C-8 (*δ*_C_ 92.5), and C-9 (*δ*_C_ 74.9). The phenylmethanone moiety was confirmed by the HMBC crossing peaks from H-19 (*δ*_H_ 8.25) and H-23 (*δ*_H_ 8.25) to C-17 (*δ*_C_ 196.4), and the connection from C-8 (*δ*_C_ 92.5) to C-17 (*δ*_C_ 196.4) was revealed by the HMBC correlation from H-9 (*δ*_H_ 4.38) to C-17 (*δ*_C_ 196.4). The HMBC correlation from H_3_-24 (*δ*_H_ 3.24) to C-8 (*δ*_C_ 92.5) confirmed the methoxy at C-8. The connection from C-10 to C-4 through C-2 and C-3 was confirmed by the HMBC correlations from H-10-OH (*δ*_H_ 5.62) to C-10 (*δ*_C_ 67.0) and C-2 (*δ*_C_ 188.5), from H-10 (*δ*_H_ 4.50) to C-2 (*δ*_C_ 188.5) and C-3 (*δ*_C_ 109.6), as well as from H_3_-16 (*δ*_H_ 1.64) to C-2 (*δ*_C_ 188.5), C-3 (*δ*_C_ 109.6), and C-4 (*δ*_C_ 196.7). In addition, the spirobicyclic moiety was suggested by the HMBC correlation from H-9 (*δ*_H_ 4.38) to C-4 (*δ*_C_ 196.7), the chemical shift of C-5 (*δ*_C_ 91.2) and the molecular formula. The *cis* configurations of 8-OCH_3_ and 9-OH were supported by the chemical shift of H-9 and the coupling constant (*δ*_H_ 4.38, *J* = 9.0 Hz) between H-9 and 9-OH [15,18]. The circular dichroism (CD) spectrum (Figure S7 in Supplementary Materials) of **1** showed negative Cotton effects at around 230, 280, and 345 nm, and positive Cotton effects at around 250 and 310 nm, which were consistent with the reported CD data for pseurotin A [18]. Thus, the structure of compound **1** was established, as shown in Figure 1, and was named cephalimysin M, its absolute configurations for C-5, C-8, and C-9 being assigned the same as those of pseurotin A. The absolute configuration of C-10 was not defined.

Compound **2** was obtained as pale yellow amorphous powder. Its molecular formula was determined as C_22_H_21_NO_8_ by HRESIMS *m/z* 450.1159 [M + Na]^+^ (calcd. for C_22_H_21_NO_8_Na 450.1159, Δmmu 0) (Figure S8 in Supplementary Materials), which accounted for thirteen degrees of unsaturation. The ^1^H, ^13^C, and HSQC NMR spectra (Figures S9–S11 in Supplementary Materials) of compound **2** showed signals of two ketone carbonyls at *δ*_C_ 193.4 (C-4) and 196.4 (C-17), one amide carbonyl at *δ*_C_ 166.4 (C-6), a mono-substituted benzene ring (*δ*_C_ 133.4, *δ*_C_ 130.4/*δ*_H_ 8.27, *δ*_C_ 128.4/*δ*_H_ 7.53, *δ*_C_ 133.9/*δ*_H_ 7.67, *δ*_C_ 128.4/*δ*_H_ 7.53, *δ*_C_ 130.4/*δ*_H_ 8.27), two sp^2^ methines (*δ*_C_ 120.1/*δ*_H_ 7.40, *δ*_C_ 108.6/*δ*_H_ 6.52), four sp^2^ quaternary carbons at *δ*_C_ 172.2 (C-2), 111.6 (C-3), 141.8 (C-10), and 163.4 (C-13), one sp^3^ methylene (*δ*_C_ 21.0/*δ*_H_ 2.76), one sp^3^ oxygenated methylene (*δ*_C_ 50.2/*δ*_H_ 4.25), one sp^3^ oxygenated methine (*δ*_C_ 75.0/*δ*_H_ 4.50), two sp^3^ oxygenated quaternary carbons (*δ*_C_ 92.1 and *δ*_C_ 92.6), and two methyls (*δ*_C_ 11.8/*δ*_H_ 1.22 and *δ*_C_ 51.7/*δ*_H_ 3.26) (Table 1). A comparison of ^1^H and ^13^C NMR data for compound **2** (Table 1) with that of the previously reported FD-838 (**4**) [15] revealed many similarities. Compound **2** had the same skeleton as that of FD-838, while methyl at C-3 of compound **4** was replaced by an oxygenated methylene in compound **2**. The mono-substituted benzene ring was identified by the COSY correlations (Figure 2, and Figure S12 in Supplementary Materials) from H-19 (*δ*_H_ 8.27) to H-23 (*δ*_H_ 8.27), through H-20 (*δ*_H_ 7.53), H-21 (*δ*_H_ 7.67), and H-22 (*δ*_H_ 7.53), and the furan side chain was characterized by COSY correlations between H-11 (*δ*_H_ 7.40) and H-12 (*δ*_H_ 6.52), and between H_2_-14 (*δ*_H_ 2.76) and H_3_-15 (*δ*_H_ 1.22), along with the HMBC correlations (Figure 2, and Figure S13 in Supplementary Materials) from H_3_-15 to C-13, and from H_2_-14 to C-12 and C-13. The lactam ring was suggested by the HMBC correlations from H-9 (*δ*_H_ 4.50) to C-5 (*δ*_C_ 92.1) and C-8 (*δ*_C_ 92.6), as well as from H-7-NH (*δ*_H_ 9.98) to C-5 (*δ*_C_ 92.1), C-8 (*δ*_C_ 92.6) and C-9 (*δ*_C_ 75.0). HMBC crossing peaks from H-19 (*δ*_H_ 8.27) and H-23 (*δ*_H_ 8.27) to C-17 (*δ*_C_ 196.4) confirmed the phenylmethanone moiety, and the connection from C-8 (*δ*_C_ 92.6) to C-17 (*δ*_C_ 196.4) was revealed by the HMBC correlation from H-9 (*δ*_H_ 4.50) to C-17 (*δ*_C_ 196.4). The methoxy at C-8 was also confirmed by the HMBC correlation from H_3_-24 (*δ*_H_ 3.26) to C-8 (*δ*_C_ 92.6). The spirobicyclic moiety was indicated by the HMBC correlation from H-9 (*δ*_H_ 4.50) to C-4 (*δ*_C_ 193.4) and the chemical shift of C-5 (*δ*_C_ 92.1). The ROESY correlations (Figure 2, and Figure S14 in Supplementary Materials) from H-9 (*δ*_H_ 4.50) to H-19 (*δ*_H_ 8.27) and H-23 (*δ*_H_ 8.27) indicated the relative configurations of C-8 and C-9. The chemical shifts of C-5, C-8, and C-9 for **2** were much closer to those of **1** and FD-838 [15], which defined the relative configurations of C-5, C-8, and C-9. The circular dichroism (CD) spectrum (Figure S15 in Supplementary Materials) of **2** showed a negative Cotton effect at around 318 nm and a positive Cotton effect at around 355 nm, which were consistent with the reported CD data for FD-838 [15]. Therefore, the structure of compound **2** was established, as shown in Figure 1, where absolute configurations were assigned the same as those of FD-838 and named cephalimysin N.

In addition to compounds **1** and **2**, known compounds were also identified in the fermentation products, such as pseurotin A (**3**) [14], FD-838 (**4**) [15], as well as four known helvolic acid derivatives, 16-*O*-propionyl-16-*O*-deacetylhelvolic acid (**5**), 6-*O*-propionyl-6-*O*-deacetylhelvolic acid (**6**) [16], helvolic acid (**7**), and 1,2-dihydrohelvolic acid (**8**) [17].

### 2.2. Biological Activity

Compounds **1**–**8** were tested against Gram positive bacteria *S. aureus* (ATCC 6538) and methicillin resistant *S. aureus* (MRSA) (ATCC 29213), Gram negative bacteria *Escherichia coli* (ATCC 11775) and *Pseudomonas aeruginosa* (ATCC 15692), BCG, and *C. albicans*. Compounds **5**–**7** showed antibacterial activities against both *S. aureus* and MRSA. Comparing the antibacterial activities of compounds **5**–**7** with the inactive analogue **8** indicated that the α,β-unsaturated ketone appears to be a key functional group for antibacterial activity (Table 2). None of the isolated compounds exhibited antimicrobial activities against *E. coli*, *P. aeruginosa*, *C. albicans* (MIC > 100 µg/mL), nor BCG (MIC > 10 µg/mL).

## 3. Materials and Methods 

### 3.1. General Experimental Procedures

The optical rotations ([α]_D_) were measured on Anton Paar MCP 200 Modular Circular Polarimeter (Austria) in a 100 × 2 mm cell at 22 °C. CD spectra were recorded on an Applied Photophysics Chirascan spectropolarimeter (UK). NMR spectra were obtained on a Bruker Avance DRX600 spectrometer with residual solvent peaks serving as references (DMSO-*d*_6_: *δ*_H_ 2.50, *δ*_C_ 39.52). High-resolution ESIMS measurements were obtained on a Bruker micrOTOF mass spectrometer by direct infusion in MeCN at 3 mL/min using sodium formate clusters as an internal calibrate. HPLC was performed using an Agilent 1200 Series separation module that was equipped with Agilent 1200 Series diode array and Agilent 1260 Series fraction collector, and Agilent SB-C18 column (250 × 9.4 mm, 5 µm).

### 3.2. Fungal Material

The *Aspergillus fumigatus* strain CUGBMF170049 was isolated from a sediment sample that was collected from the Bohai Sea, China and grown on a potato dextrose agar plate at 28 °C. This strain was identified as *Aspergillus fumigatus* based on DNA sequence analysis of its internal transcribed spacer (ITS) region (Figure S16) (GenBank accession number MK453215) using a conventional primer pair of ITS5 (5′-GGAAGTAAAAGTCGTAACAAGG-3′) and ITS4 (5′-TCCTCCGCTTATTGATATGC-3′). 

### 3.3. Fermenttion and Extraction

A small spoonful of *Aspergillus fumigatus* (CUGBMF170049) spores growing on a potato dextrose agar slant was inoculated into four 250 mL conical flasks, each containing 40 mL of liquid medium consisting of potato infusion (20%), glucose (2.0%), artificial sea salt (3.5%), and distilled water. The flasks were incubated at 28 °C for 3 d on a rotary shaker at 160 rpm. An aliquot (5 mL) of the resultant seed culture was inoculated into teen 1 L conical flasks, with each containing solid medium consisting of rice (120 g) and artificial seawater (3.5%; 80 mL), and the flasks were incubated stationary for 30 days at 28 °C. The cultures were extracted three times by EtOAc:MeOH (80:20), and the combined extracts were reduced to dryness in vacuo and the residue was partitioned between EtOAc and H_2_O. Subsequently, the EtOAc layer was dried in vacuo to yield a dark residue (11.3 g). 

### 3.4. Isolation and Purification

The EtOAc fraction was fractionated by a reduced pressure silica gel chromatography (50 × 80 mm column, TLC H silica) using a stepwise gradient of 50–100% hexane/CH_2_Cl_2_ and then 0–100% MeOH/CH_2_Cl_2_ to afford 15 fractions. Fraction C was fractionated on a Sephadex LH-20 column (600 × 30 mm) while using an isocratic elution of hexane:CH_2_Cl_2_:MeOH (5:5:1) to give five subfractions (F1–F5). Subfraction F3 (102.3 mg after drying in vacuo) was further fractionated by HPLC (Agilent Zorbax SB-C18 250 × 9.4 mm, 5 μm column, 2.0 mL/min, isocratic 65% MeOH/H_2_O) to yield FD-838 (**4**; *t*_R_ 10.4 min, 3.3 mg). Fraction J was fractionated on a Sephadex LH-20 column (600 × 30 mm) using an isocratic elution of CH_2_Cl_2_:MeOH (2:1), to give four subfractions (F1–F4). Subfraction F1 was further fractionated by HPLC (Agilent Zorbax SB-C18 250 × 9.4 mm, 5 μm column, 2.0 mL/min, isocratic 65% MeOH/H_2_O) to yield helvolic acid (**7**, t_R_ 10.8 min, 1.3 mg), 16-*O*-propionyl-16-*O*-deacetylhelvolic acid (**5,** *t*_R_ 11.9 min, 1.2 mg), and 6-*O*-propionyl-6-*O*-deacetylhelvolic acid (**6**, *t*_R_ 12.4 min, 1.4 mg). Fraction K was fractionated on a Sephadex LH-20 column (600 × 30 mm) using an isocratic elution of CH_2_Cl_2_:MeOH (2:1) to give five subfractions (F1–F5). Subfraction F3 was further fractionated by HPLC (Agilent Zorbax SB-C18 250 × 9.4 mm, 5 μm column, 2.0 mL/min, isocratic 65% MeOH/H_2_O) to yield 1,2-dihydrohelvolic acid (**8**, t_R_ 13.9 min, 1.6 mg). Fraction L was fractionated on a Sephadex LH-20 column (600 × 30 mm) using an isocratic elution of CH_2_Cl_2_:MeOH (2:1) to give five subfractions (F1–F5). Subfraction F4 was further fractionated by an ODS column, which was eluted by a stepwise gradient (0–100% MeOH/H_2_O) to afford five subfractions (F1–F5). Subfraction F4 was further fractionated by HPLC (Agilent Zorbax SB-C18 250 × 9.4 mm, 5 μm column, 2.0 mL/min, isocratic 65% MeOH/H_2_O) to yield pseurotin A (**3**, t_R_ 7.1 min, 3.2 mg), cephalimysins M (**1**, *t*_R_ 12.0 min, 1.5 mg), and N (**2**, *t*_R_ 8.9 min, 3.6 mg).

#### 3.4.1. Cephalimysin M (**1**)

Pale yellow amorphous powder; [α]${}_{D}^{22}$ –21.3 (MeOH, 0.1); UV (MeOH) λ_max_ (logε) 196 (4.43), 254 (4.14), 277(3.96) nm; (+)-ESIMS *m*/*z* 418.1 [M + H]^+^; (+)-HRESIMS *m*/*z* 440.1684 [M + Na]^+^ (calcd. for C_22_H_27_NO_7_Na 440.1680); ^1^H and ^13^C NMR data: See Table 1.

#### 3.4.2. Cephalimysin N (**2**)

Pale yellow amorphous powder; [α]${}_{D}^{22}$ –21.5 (MeOH, 0.1); UV (MeOH) 197 (4.43), 252 (4.12), 329(3.56) nm; (+)-ESIMS *m*/*z* 428.0 [M + H]^+^; (+)-HRESIMS *m*/*z* 450.1159 [M + Na]^+^ (calcd. for C_22_H_21_NO_8_Na 450.1159); ^1^H and ^13^C NMR data: See Table 1.

### 3.5. Antimicrobial Assays

The antimicrobial assays were performed according to the Antimicrobial Susceptibility Testing Standards that were outlined by the Clinical and Laboratory Standards Institute (CLSI) against *S. aureus* ATCC 6538, MRSA ATCC 29213, *E. coli* ATCC 11775, *P. aeruginosa* ATCC 15692, and *C. albicans* ATCC 10231 based on a 96 well microplate format in liquid growth. Briefly, the bacteria from glycerol stocks was inoculated on LB agar plate and cultured overnight at 37°C. The glycerol stock of *C. albicans* was prepared on Sabouraud dextrose agar at 28 °C for 24 h. Afterwards, single colonies were picked and adjusted to approximately 10^4^ CFU/mL with Mueller–Hinton Broth as bacterial suspension and with RPMI 1640 media as fungal suspension. 2 μL of two-fold serial dilution of each compound (in DMSO) were added to each row on 96-well microplate, containing 78 μL of bacterial or fungal suspension in each well. (Vancomycin and Ciprofloxacin were used as positive controls; Amphotericin B was used as positive for fungi; DMSO as negative control). The 96-well plate was aerobically incubated at 37 °C for 16 h. The 96-well plate of antifungal was aerobically incubated at 35 °C for 24 h. Here, MIC is defined as the minimum concentration of compound at which no bacterial growth is observed.

### 3.6. Anti-Bacillus Calmette Guérin (BCG) Assay

The anti-BCG assay was carried out by using a constitutive GFP expression strain (pUV3583c-GFP), according to previous published procedure (isoniazid was used as positive control with MIC value of 0.05 µg/mL) [19]. The concentrations for the tested compounds were from 0.156 to 10 µg/mL by using two-fold diluted solutions.

## 4. Conclusions

As part of our ongoing research program to discover novel secondary metabolites from the marine environment, eight compounds were isolated from the rice solid medium culture of the marine derived fungus CUGBMF170049 isolated from a sediment sample that was collected from the Bohai Sea, China. Two novel compounds (**1** and **2**) were isolated and characterized along with the previously reported analogues pseurotin A (**3**) and FD-838 (**4**), as well as four known helvolic acid derivatives, namely 16-*O*-propionyl-16-*O*-deacetylhelvolic acid (**5**), 6-*O*-propionyl-6-*O*-deacetylhelvolic acid (**6**), helvolic acid (**7**), and 1,2-dihydrohelvolic acid (**8**). All of the structures were confirmed by detailed analysis of the spectroscopic data. Compounds **5**–**7** showed antibacterial activity against both *S. aureus* and MRSA. Aanalogue **8** did not exhibit antibacterial activities that indicated that the α,β-unsaturated ketone of **5**–**7** is the key functional group for antibacterial activity. Structurally, cephalimysins M (**1**) and N (**2**) belong to a family of rare natural products with diverse biological activities, which contain an unusual spiro-heterocyclic γ-lactam core. To the best of our knowledge, 28 natural products of this family have been reported, including pseurotin A [14], 8-*O*-demethylpseurotin A [20] pseurotins A1 and A2 [18,21], pseurotins B – E [22], pseurotins F1 and F2 [23], 14-norpseurotin A [24] synerazol [25], azaspirene [26], azaspirofurans A and B [27], and FD-838 and cephalimysins A–L [15,28,29]. **2** is the first cephalimysin analogue where the methyl of C-16 was oxidized to hydroxymethyl. The current research diversifies the structures of this class of natural products. 

## Figures and Tables

**Table 1:** NMR data for **1** and **2** (DMSO-*d*_6_).

Position	1		2	
	*δ*_C_, Type	*δ*_H,_ mult (*J* in Hz)	*δ*_C_, Type	*δ*_H,_ mult (*J* in Hz)
2	188.5, C		172.2, C	
3	109.6, C		111.6, C	
4	196.7, C		193.4, C	
5	91.2, C		92.1, C	
6	166.4, C		166.4, C	
8	92.5, C		92.6, C	
9	74.9, CH	4.38, d (9.0)	75.0, CH	4.50, s
10	67.0, CH	4.50, td (7.2, 5.4)	141.8, C	
11	34.4, CH_2_	1.64, m (overlap)	120.0, CH	7.40, d (3.6)
12	24.1, CH_2_	1.37, m	108.6, CH	6.52, d (3.6)
13	31.1, CH_2_	1.24, m	163.4, C	
14	22.0, CH_2_	1.24, m	21.0, CH_2_	2.76, q (7.8)
15	13.9, CH_3_	0.84, t (7.2)	11.8, CH_3_	1.22, t (7.8)
16	5.4, CH_3_	1.64, s	50.2, CH_2_	4.25, s
17	196.4, C		196.4, C	
18	133.4, C		133.4, C	
19	130.3, CH	8.25, dd (8.4, 1.2)	130.4, CH	8.27, d (7.2)
20	128.4, CH	7.53, ddd (8.4, 8.4, 1.2)	128.4, CH	7.53, dd (7.2, 7.2)
21	133.9, CH	7.64, dddd (8.4, 8.4, 1.2, 1.2)	133.9, CH	7.67, dd (7.2, 7.2)
22	128.4, CH	7.53, ddd (8.4, 8.4, 1.2)	128.4, CH	7.53, dd (7.2, 7.2)
23	130.3, CH	8.25, dd (8.4, 1.2)	130.4, CH	8.27, d (7.2)
24	51.7, CH_3_	3.24, s	51.7, CH_3_	3.26, s
7-NH		9.90, s		9.98, s
9-OH		6.34, d (9.0)		
10-OH		5.62, d (5.4)		

**Table 2:** Antimicrobial activities of **1**–**8** (µg/mL).

Compounds	*S. aureus* ^a^	MRSA ^a^
**1**	>100	>100
**2**	>100	>100
**3**	>100	>100
**4**	>100	>100
**5**	12.5	25
**6**	6.25	12.5
**7**	0.78	0.78
**8**	>100	>100

^a^ Vancomycin was used as positive control with MIC value of 0.78 µg/mL.

## Supplementary Materials

The following are available online at https://www.mdpi.com/1660-3397/17/5/289/s1, Figures S1–S15: The HRESIMS, UV, 1 D, 2D NMR, and CD spectra of compounds **1** and **2**, Figure S16 the phylogenetic tree of strain CUGBMF170049.
