# Supplementary material for: Two New Spiro-Heterocyclic γ-Lactams from A Marine-Derived Aspergillus fumigatus Strain CUGBMF170049
Source: Mar Drugs. 2019 May 14;17(5):289. doi: 10.3390/md17050289 (PMC6562449; doi:10.3390/md17050289)
Supplement: Supplementary file 1 [file marinedrugs-17-00289-s001.pdf]

Supplementary Materials

Two new spiro-heterocyclic γ-lactams from a marine-derived *Aspergillus fumigatus* strain CUGBMF170049

Xiuli Xu [1*], Jiahui Han [1,2], Yanan Wang[1], Rui Lin [1], Haijin Yang[1], Jiangpeng Li [1], Shangzhu Wei [1] ,Steven W. Polyak [3] and Fuhang Song [2] *

[1] School of Ocean Sciences, China University of Geosciences, Beijing 100083, China

[2] CAS Key Laboratory of Pathogenic Microbiology and Immunology, Institute of Microbiology, Chinese Academy of Sciences, Beijing 100101, China

[3] School of Pharmacy and Medical Sciences, University of South Australia, Adelaide 5000, Australia

Content

Figure S1. HRESIMS for compound **1** 3

Figure S2. ^{1}H NMR and UV spectra (600 MHz, DMSO-d_6) of **1** 3

Figure S3. ^{13}C NMR spectrum (150 MHz, DMSO-d_6) of **1** 4

Figure S4. HSQC spectrum (600 MHz, DMSO-d_6) of **1** 4

Figure S5. ^{1}H -^{1}H COSY spectrum (600 MHz, DMSO-d_6) of **1** 5

Figure S6. HMBC spectrum (600 MHz, DMSO-d_6) of **1** 5

Figure S7. CD spectra of **1** and **3** (MeOH) 6

Figure S8. HRESIMS for compound **2** 6

Figure S9. ^{1}H NMR and UV spectra (600 MHz, DMSO-d_6) of **2** 7

Figure S10. ^{13}C NMR spectrum (150 MHz, DMSO-d_6) of **2** 7

Figure S11. HSQC spectrum (600 MHz, DMSO-d_6) of **2** 8

Figure S12. ^{1}H -^{1}H COSY spectrum (600MHz, DMSO-d_6) of **2** 8

Figure S13. HMBC spectrum (600 MHz, DMSO-d_6) of **2** 9

Figure S14. ROESY spectrum (600 MHz, DMSO-d_6) of **2** 9

Figure S15. CD spectrum of **2** (MeOH) 10

Figure S16. Neighbor-joining phylogenetic tree of strain CUGBMF170049 10

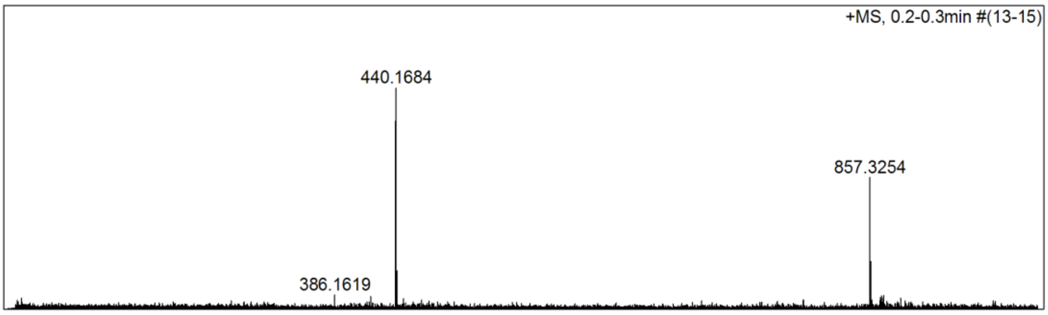

Figure S1. HRESIMS for compound **1**

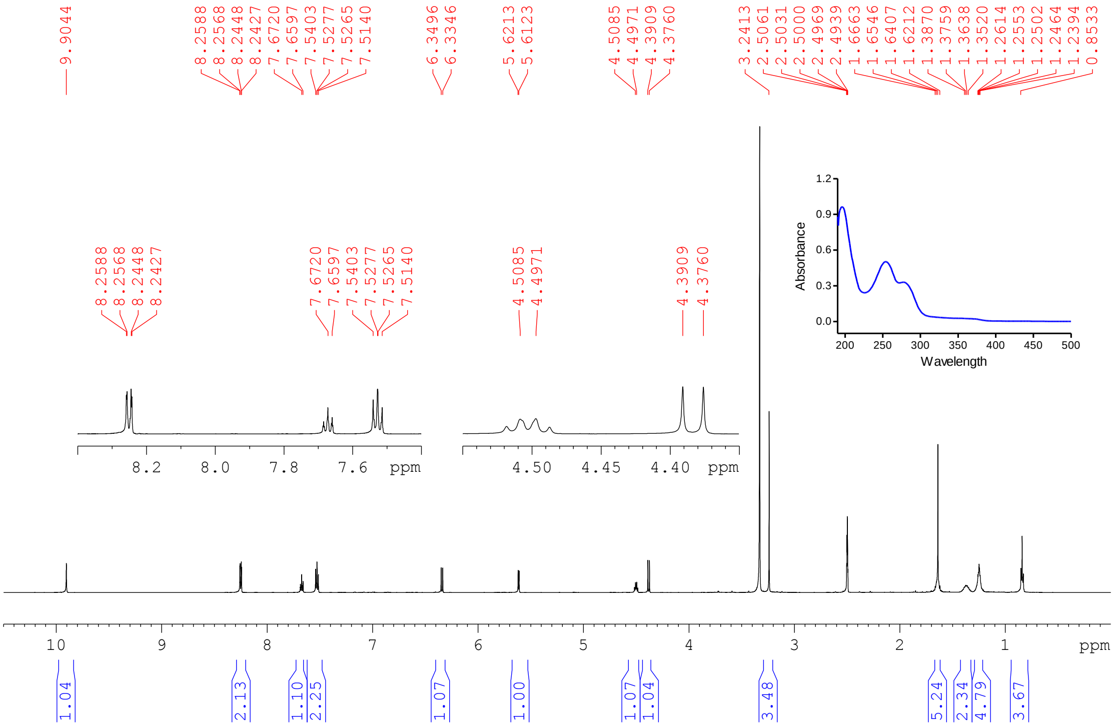

Figure S2. ^{1}H NMR spectrum (600 MHz, DMSO-d_6) of **1**

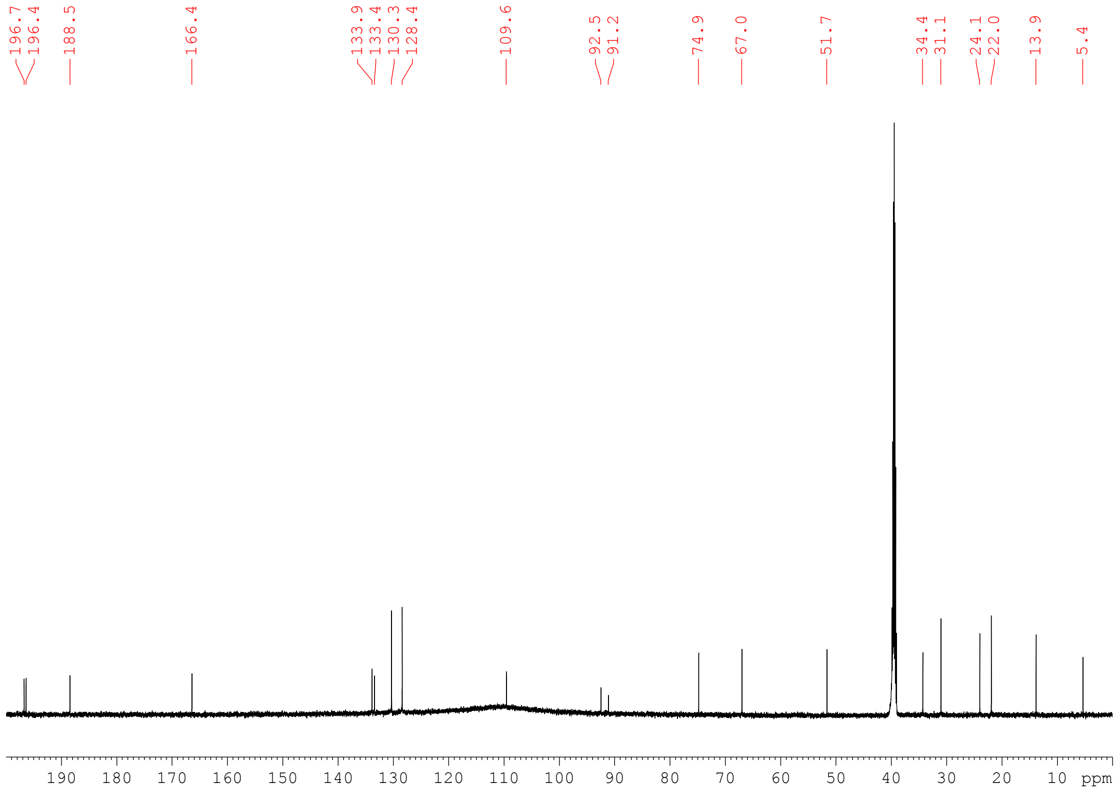

Figure S3. ^{13}C NMR spectrum (150 MHz, DMSO-d_6) of **1**

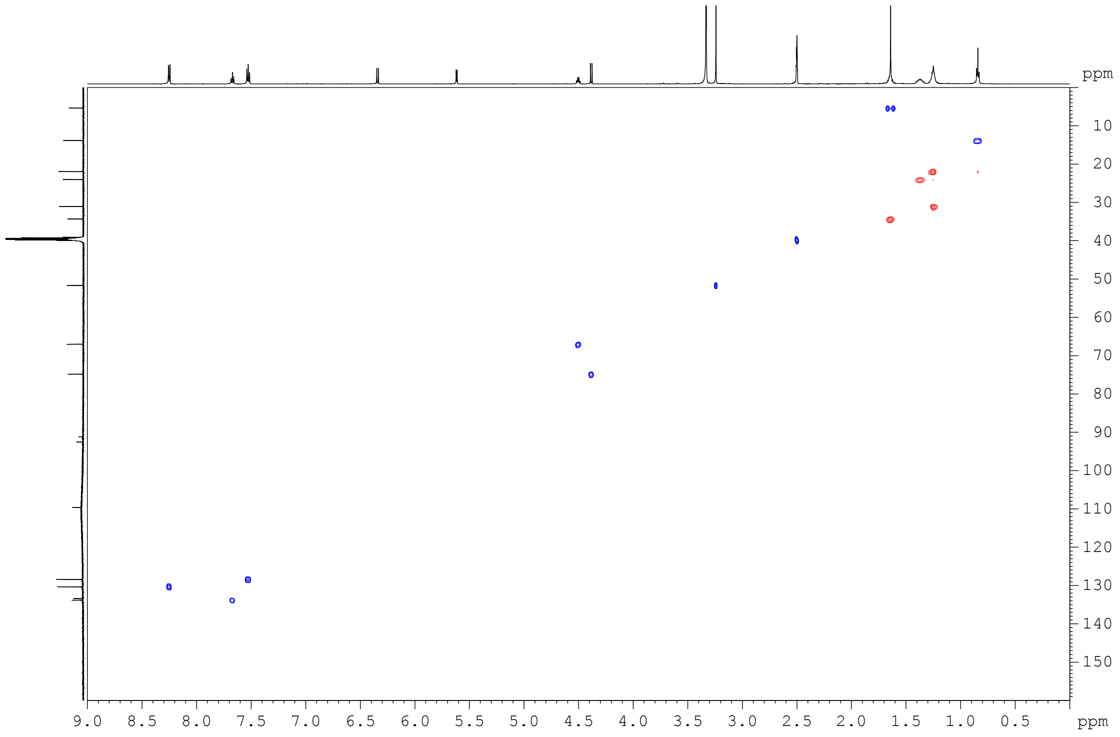

Figure S4. HSQC spectrum (600 MHz, DMSO-d_6) of **1**

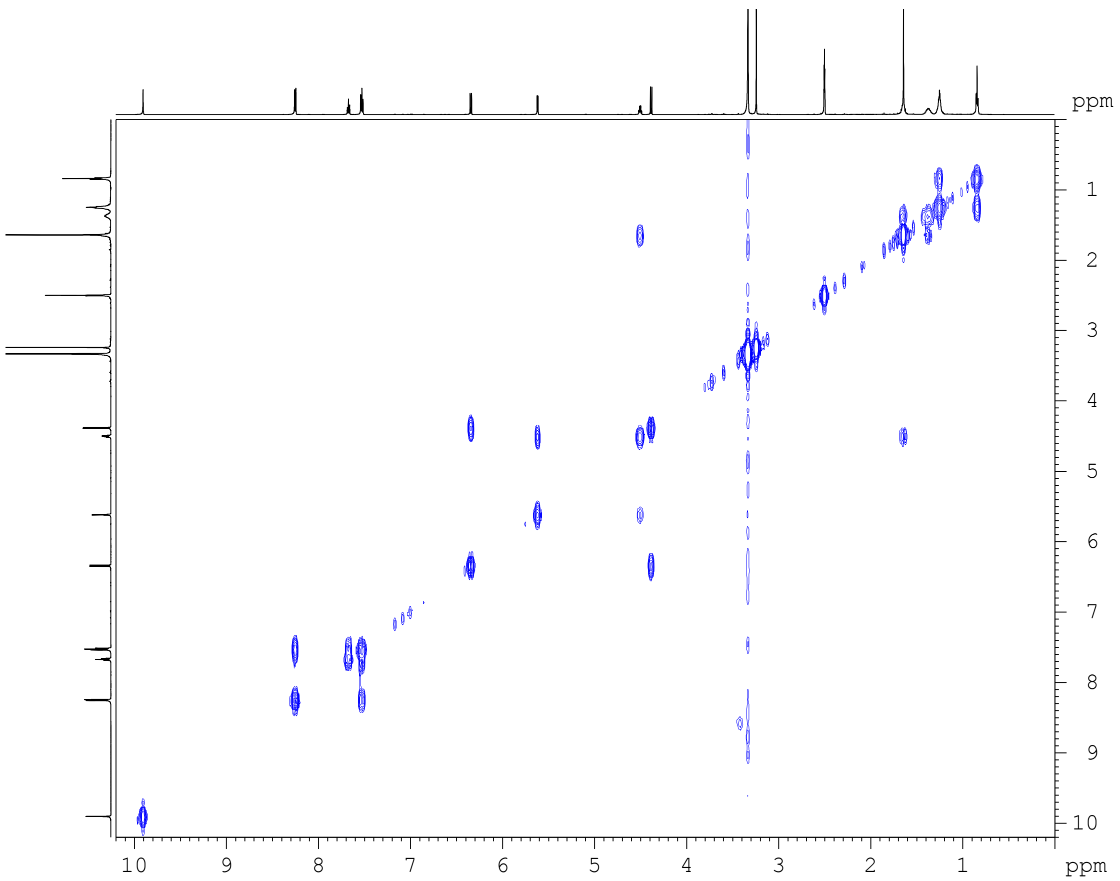

Figure S5. ^{1}H -^{1}H COSY spectrum (600 MHz, DMSO-d_6) of **1**

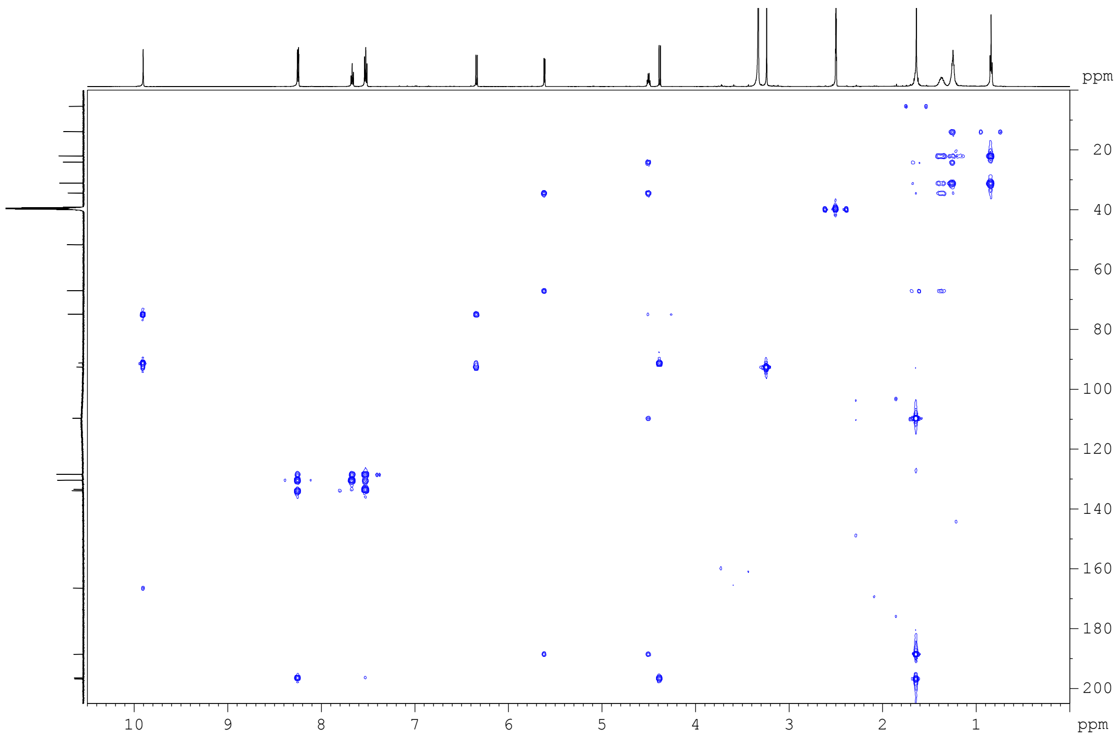

Figure S6. HMBC spectrum (600 MHz, DMSO-d_6) of **1**

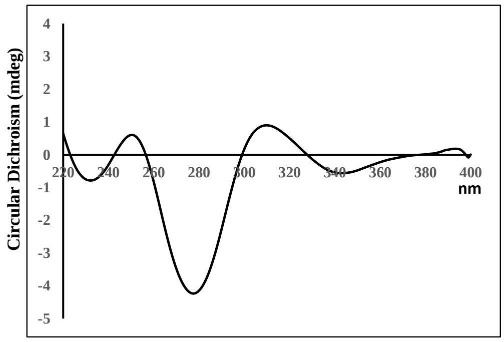

1

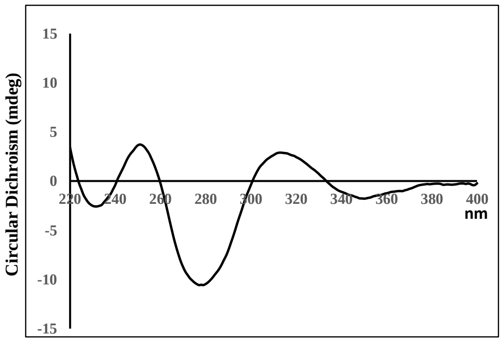

3

Figure S7. CD spectra of **1** and **3** (MeOH)

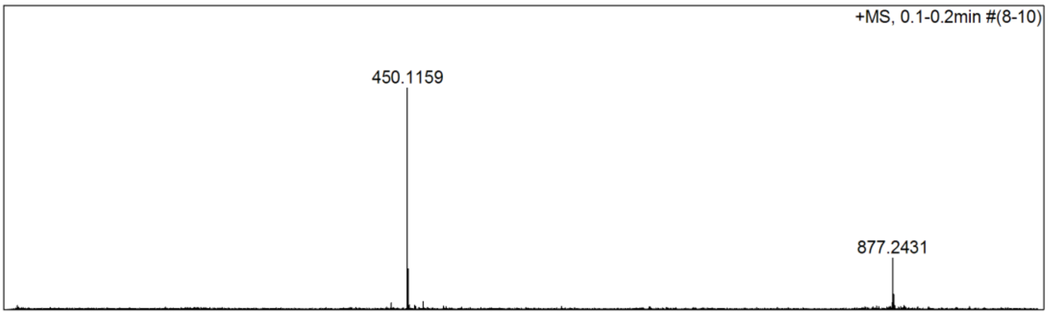

Figure S8. HRESIMS for compound **2**

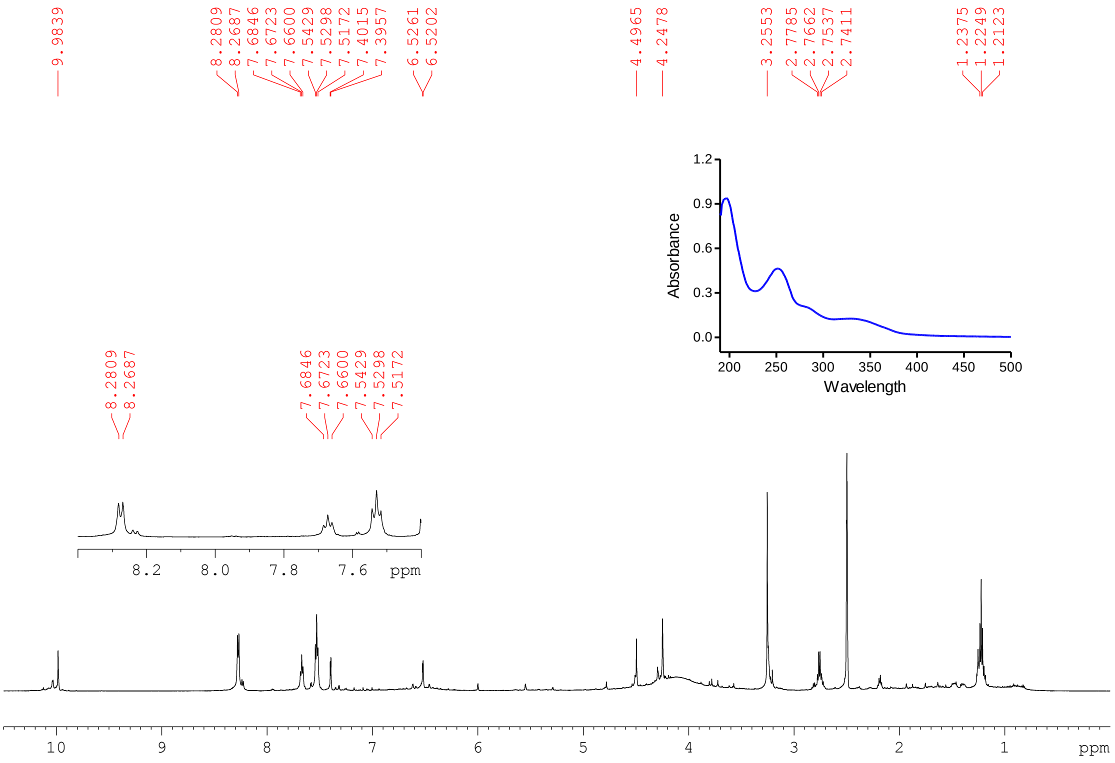

Figure S9. ^{1}H NMR spectrum (600 MHz, DMSO-d_6) of **2**

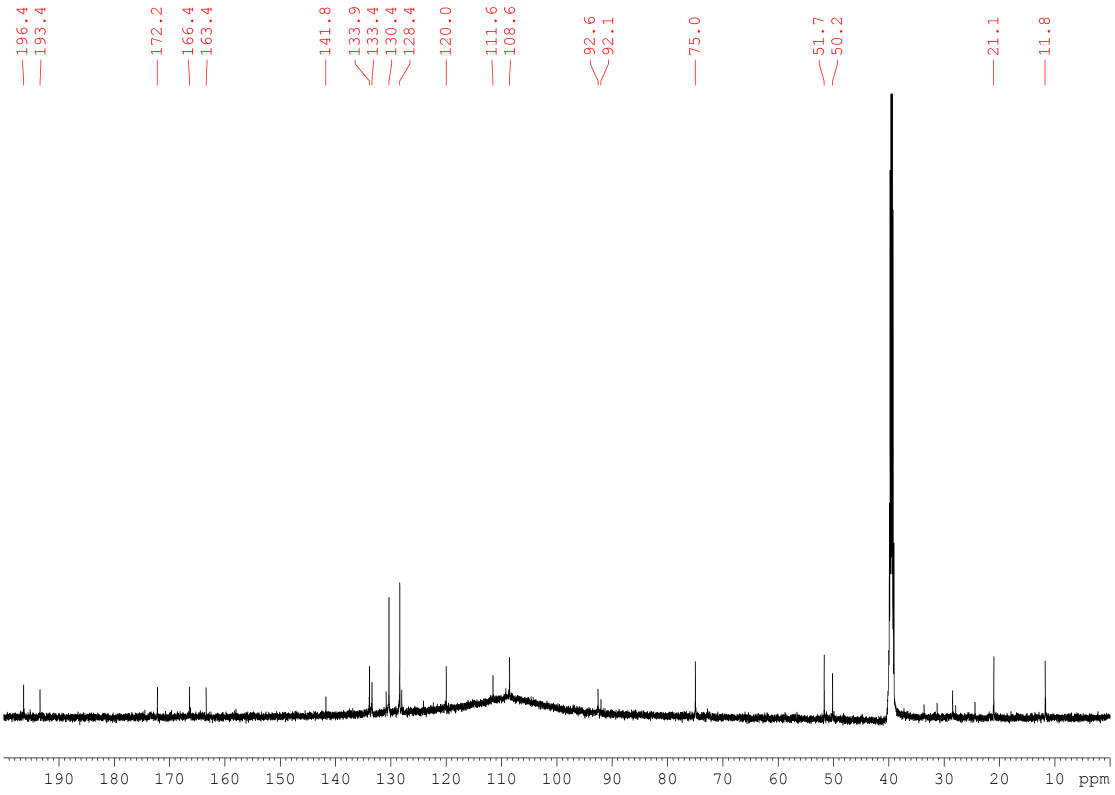

Figure S10. ^{13}C NMR spectrum (150 MHz, DMSO-d_6) of **2**

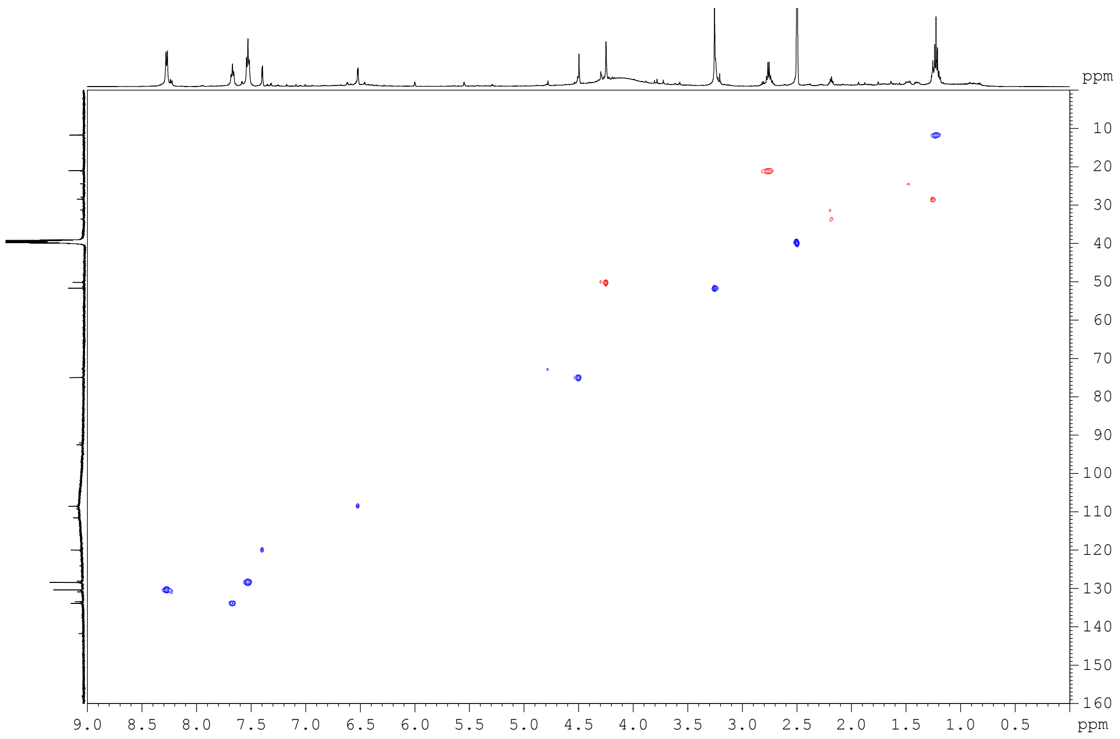

Figure S11. HSQC spectrum (600 MHz, DMSO-d_6) of **2**

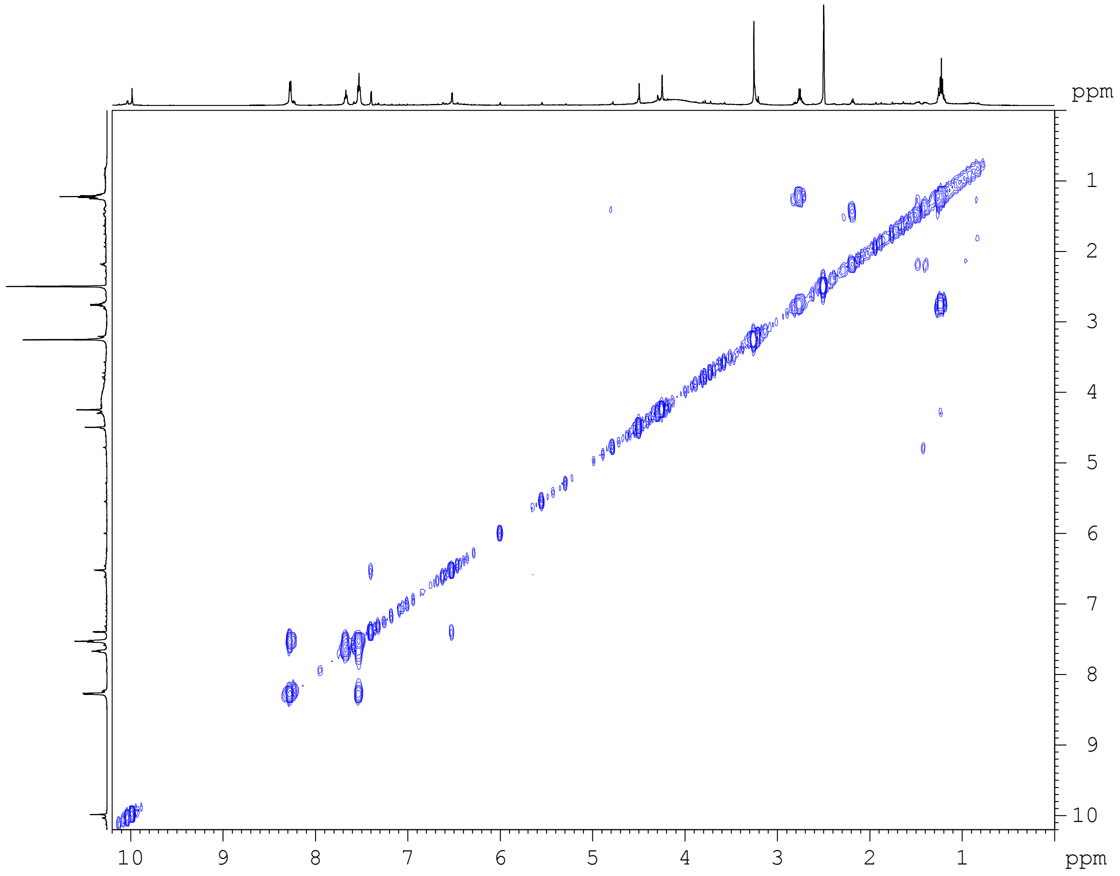

Figure S12. ^{1}H -^{1}H COSY spectrum (600MHz, DMSO-d_6) of **2**

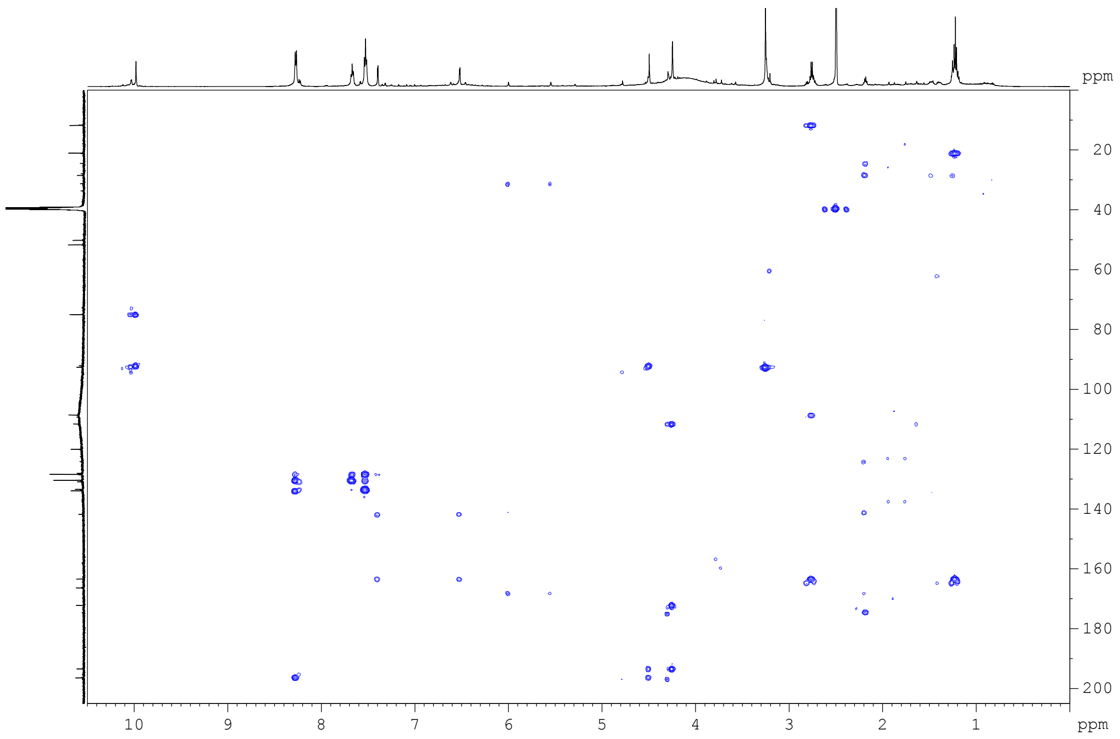

Figure S13. HMBC spectrum (600 MHz, DMSO-d_6) of **2**

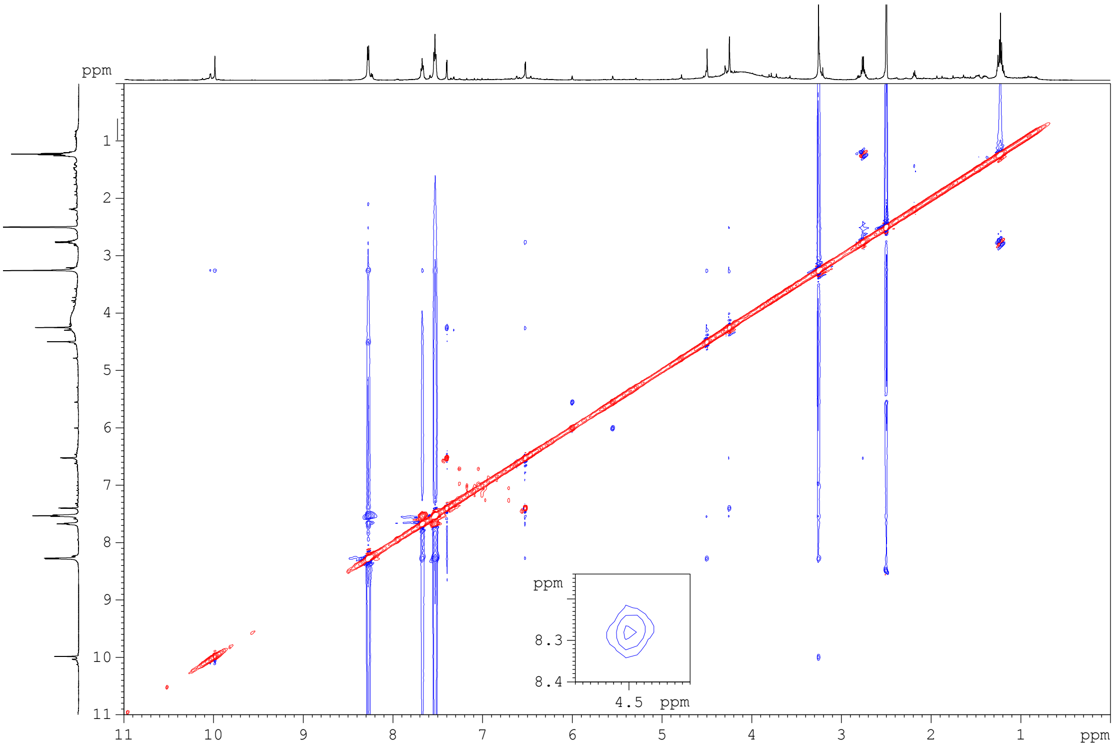

Figure S14. ROESY spectrum (600 MHz, DMSO-d_6) of **2**

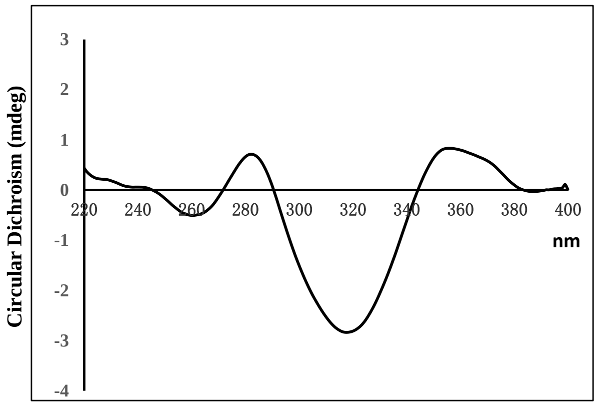

2

Figure S15. CD spectrum of **2**

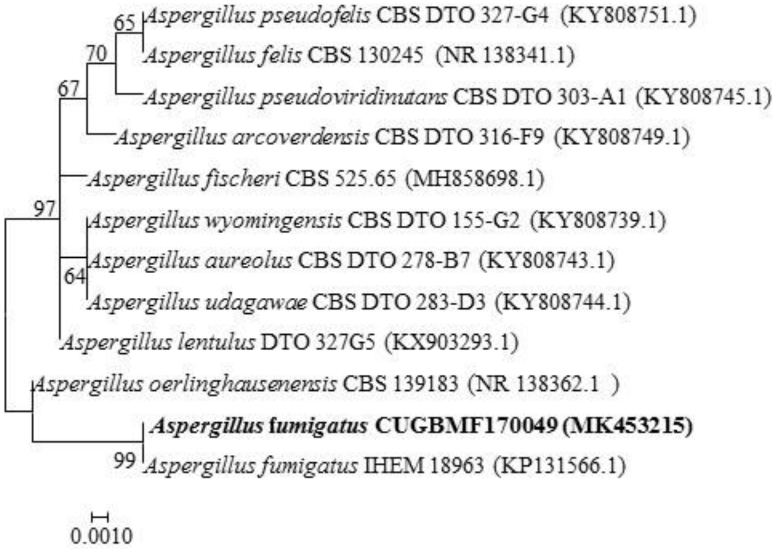

Figure S16. Neighbor-joining phylogenetic tree of strain CUGBMF170049

Numbers at nodes indicate levels of bootstrap support (%) based on a neighbor joining analysis of 1,000 resampled datasets; only values >50 % are given. NCBI accession numbers are given in parentheses. Bar 0.001 nucleotide substitutions per site
